# Built Environment: Turn Up the Heat for Respiratory Health

**DOI:** 10.1289/ehp.116-a291

**Published:** 2008-07

**Authors:** Adrian Burton

In the dog days of summer it’s hard to think about turning up the thermostat, but keep one thing in mind as the cooler months approach: people with chronic obstructive pulmonary disease (COPD) whose houses are cold in cold weather are more likely to suffer poorer respiratory health than those whose houses are warmer, according to a study published online 26 March 2008 ahead of print in the *European Journal of Public Health*. Turning up the heat might therefore lead to more benefits than just warm feet.

“This is the first time a direct relationship has been found between the number of hours a house is warm and respiratory health status—in this case that of patients with COPD,” explains first author Liesl Osman, a research development advisor with the Nuffield Department of Clinical Medicine, Oxford University. “And it would seem that this relationship is most marked for smokers.”

The researchers monitored the temperature of the living rooms and bedrooms of 148 COPD patients in Aberdeen, Scotland, for 1 week between October and May (all of which are cold months in Scotland). They tracked how often each living room was kept at a temperature of at least 21°C (69.8°F)—which the U.K. government recommends be maintained for at least 9 hours per day for maximal energy efficiency—and how long each bedroom maintained a temperature of at least 18°C (64.4°F). The subjects also completed questionnaires to determine their respiratory and general health status.

Overall, people who lived in homes in which the living room temperature was more typically at least 21°C for at least 9 hours per day had significantly better respiratory health (that is, fewer exacerbations of their underlying COPD or respiratory infections) than subjects who lived in homes where this temperature was maintained less often. “Unfortunately,” Osman adds, “we also saw that over fifty percent of the households involved in this study were colder than recommended.” No association was seen between respiratory health scores and bedroom temperatures.

In a post hoc analysis, the protective effect of warmth was statistically significant for smokers but did not reach significance for nonsmokers. “Prolonged cold weakens our resistance to infection,” explains Osman. “Perhaps this is accentuated in smokers with COPD, resulting in overall poorer respiratory health.”

Philippa Howden-Chapman, director of the Housing and Health Research Programme at the University of Otago in Wellington, New Zealand, also points out that cold homes could be the cause of poorer health in people with other underlying diseases. “[We have] shown that increasing warmth leads to improvements in health for most respiratory diseases, and our recently completed Housing, Heating and Health Study found warmer temperatures improved the respiratory symptoms of children with asthma,” she says. The literature also suggests that coronary conditions are affected by indoor temperatures, particularly in temperate countries.

“The key question that needs to be answered is whether improvements in home heating will actually bring out the benefits to health [that may be] predicted using data from this cross-sectional study,” remarks Anna Hansell, a clinical fellow at Imperial College London. Howden-Chapman agrees: “This is a cross-sectional study, so causal conclusions really shouldn’t be drawn from it, though the results are very interesting. The next step would be to [look at] daily measures to see whether short-term exposure to cold influences COPD symptoms. This would also help determine the precision of [these] long-term measures.”

The United Kingdom has social programs to help people with low incomes or poorer housing increase the energy efficiency of their homes, which can help conserve heat. Given today’s sky-high oil and gas prices, the results of this study could mean such schemes are more needed than ever.

